# Compositional alterations in immune cells from obese mice impair anti-tumor cytotoxicity in pancreatic ductal adenocarcinoma

**DOI:** 10.29219/fnr.v70.13589

**Published:** 2026-08-28

**Authors:** Inae Jeong, Ok-Kyung Kim

**Affiliations:** 1 Division of Food and Nutrition, Chonnam National University, Gwangju, Republic of Korea; 2 Human Ecology Research Institute, Chonnam National University, Gwangju, Republic of Korea

**Keywords:** antitumor immunity, obesity, pancreatic ductal adenocarcinoma, tumor microenvironment, immunity, immune checkpoint

## Abstract

**Background:**

Obesity is a major risk factor for pancreatic ductal adenocarcinoma (PDAC) and is associated with poor prognosis. Although metabolic and inflammatory disturbances linking obesity to PDAC progression are well-documented, the impact of obesity-induced immune dysfunction on the tumor microenvironment remains unclear.

**Objective:**

This study aimed to elucidate the effects of obesity on the anti-tumor immune response in pancreatic cancer.

**Design:**

Splenocytes isolated from mice fed a normal chow (NC) or high-fat diet (HFD) for 12 weeks were analyzed by flow cytometry. After co-culture with Panc02 cells, both splenocytes and PDAC cells (Panc02) were subjected to flow cytometry.

**Results:**

Splenocytes derived from HFD-fed mice exhibited a markedly reduced abundance of CD8^+^ T and natural killer (NK) cells compared with those from NC-fed mice, despite unchanged cytokine-producing capacity. Co-culture experiments with Panc02 revealed that HFD-derived splenocytes significantly diminished tumor cell apoptosis, coinciding with reduced frequencies of CD8^+^ T and NK cells. Notably, while PD-1 expression on CD8^+^ T and NK cells remained stable, PD-L1 expression on Panc02 cells significantly decreased following co-culture with HFD-derived splenocytes.

**Conclusions:**

These findings indicate that obesity impairs anti-tumor immunity in PDAC by reducing the abundance of cytotoxic lymphocytes, thereby limiting tumor cell apoptosis and PD-L1 induction.

## Popular scientific summary

- Splenocytes from obese mice showed a decrease in CD8^+^ T and NK cells.
- Splenocytes from obese mice reduced tumor cell apoptosis and PD-L1 induction in Panc02 cells.
- Obesity impairs anti-tumor immunity in PDAC by reducing cytotoxic lymphocyte abundance.

Pancreatic cancer is one of the most lethal malignancies, with an overall 5-year survival rate of less than 10%. This statistic is attributed to late diagnosis, aggressive biological behavior, and resistance to standard therapies. Among the various types of pancreatic cancer, pancreatic ductal adenocarcinoma (PDAC) accounts for most cases and is particularly challenging to treat. Despite advancements in surgical techniques, chemotherapy, and targeted therapies, the prognosis for PDAC remains poor, highlighting the urgent need to identify modifiable risk factors and underlying mechanisms that contribute to disease onset and progression (1).

Among the various risk factors associated with PDAC, obesity has emerged as a significant determinant of both cancer incidence and mortality (2–4). In a systematic review and meta-analysis of prospective studies conducted by Aune et al. (4), an increased risk of pancreatic cancer correlated with higher body mass index, waist circumference, and waist-to-hip ratio values. Previous mechanistic studies have generally attributed the association between obesity and pancreatic cancer to systemic metabolic alterations. Obesity-induced adipose tissue expansion leads to chronic low-grade inflammation, aberrant adipokine secretion, and insulin resistance, all of which create a tumor-permissive systemic environment (5, 6). While these processes have been proposed as primary drivers linking obesity to tumorigenesis, the impact of obesity-induced changes in immune cells on the tumor microenvironment (TME) and tumor–immune interactions remains unclear.

Recent evidence indicates that obesity-induced immune dysfunction encompasses both systemic and local immune compartments, characterized by impaired CD8^+^ T cell function, reduced natural killer (NK) cell frequency, and the emergence of regulatory or exhausted immune phenotypes (7–9). Considering that effective anti-tumor immunity relies on cytotoxic lymphocytes, obesity-driven alterations in these populations may represent a crucial yet underexplored mechanism contributing to impaired immune surveillance in pancreatic cancer.

This study aimed to investigate the effects of obesity on the anti-tumor activity of immune cells using a co-culture system involving PDAC cells (Panc02) and splenocytes derived from mice fed either a normal chow (NC) diet or a high-fat diet (HFD). By examining apoptosis induction in cancer cells, PD-L1 expression, and the functional dynamics of CD8^+^ T and NK cells, we elucidated the mechanisms by which obesity compromises anti-tumor immunity. These findings provide novel insights into how obesity alters the TME through immune cell modifications, thereby attenuating immune-mediated tumor suppression.

## Materials and methods

### Animals

Six-week-old C57BL/6J male mice were obtained from Central Lab Animal, Inc. (Seoul, Korea). The mice were housed under controlled environmental conditions (22–25°C, 50% humidity) with a 12-h light/dark cycle. Following a 1-week acclimatization period, the mice were randomly assigned to either a NC group or a HFD group (*n* = 8 mice). Randomization was performed using a computer-generated random sequence to ensure unbiased group allocation. To minimize potential confounding factors, cages were rotated weekly within the animal room, and handling and sampling were performed in a randomized order. All animal study protocols were approved by the Institutional Animal Care and Use Committee of Chonnam National University (CNU IACUC-YB-2022-19), and the animals were maintained in accordance with the university’s Guidelines for Animal Experiments.

### Cell culture

Panc02, a murine PDAC cell line, was cultured in Dulbecco’s Modified Eagle Medium supplemented with 10% fetal bovine serum (FBS) and 1% penicillin–streptomycin. Mouse splenocytes were isolated by dissociating freshly harvested spleens through a 40-µm cell strainer, followed by red blood cell lysis. The isolated splenocytes were subsequently washed and resuspended in RPMI-1640 medium containing 10% FBS and 1% penicillin–streptomycin. For co-culture, splenocytes were seeded onto Panc02 cells at a ratio of 250:1 and incubated for 42 h prior to collection for analysis. All cultures were maintained in a humidified atmosphere of 5% CO_2_ at 37°C.

### Flow cytometry

Dead cells were excluded using a Live/Dead Fixable Aqua Dead Cell Stain Kit (Thermo Fisher Scientific) following the manufacturer’s protocol. Intracellular cytokines were stained after treating the cells with a fixation/permeabilization buffer (Enzo Life Sciences). The following fluorescent-conjugated antibodies were utilized: tumor necrosis factor alpha (TNF-α)–fluorescein isothiocyanate (FITC) (#11-7321-82; Thermo Fisher Scientific), transforming growth factor beta (TGF-β)–phycoerythrin (PE) (#141404; BioLegend), interleukin-12 (IL-12)–allophycocyanin (APC) (#554480; BD Biosciences), cluster of differentiation (CD)4–FITC (#1540-02; SouthernBiotech), CD154–eFluor 450 (#48-1541-82; Thermo Fisher Scientific), CD3–eFluor 450 (#48-0032-82; Thermo Fisher Scientific), CD8–PE/cyanine 5.5 (CY5.5) (#1550-16; SouthernBiotech), interferon gamma (IFN-γ)–APC (#17-7311-82; Thermo Fisher Scientific), NK cell antigen 1.1 (NK1.1)–FITC (#11-5941-82; Thermo Fisher Scientific), CD107a–PE (#12-1071-82; Thermo Fisher Scientific), programmed death-ligand 1 (PD-L1)–APC (#LS-C764395; LSBio), and PD-1–APC–eFluor 780 (#47-9985-82; Thermo Fisher Scientific). Apoptosis was assessed using a FITC Annexin V Apoptosis Detection Kit I (BD Biosciences). Data were acquired using a CytoFLEX flow cytometer (Beckman Coulter, CA, USA) and analyzed with CytExpert software (Beckman Coulter).

### Statistical analysis

All data are presented as the mean ± SD. To evaluate the effects of diet-induced splenocyte inoculation under defined co-culture conditions, differences between the two groups were analyzed using Student’s *t*-test in IBM SPSS Statistics for Windows (version 23.0; IBM Corp., Armonk, NY, USA). Statistical significance was set at *P* < 0.05.

## Results

### HFD feeding alters splenocyte composition but not cytokine profiles in mice

**Fig. 1:**
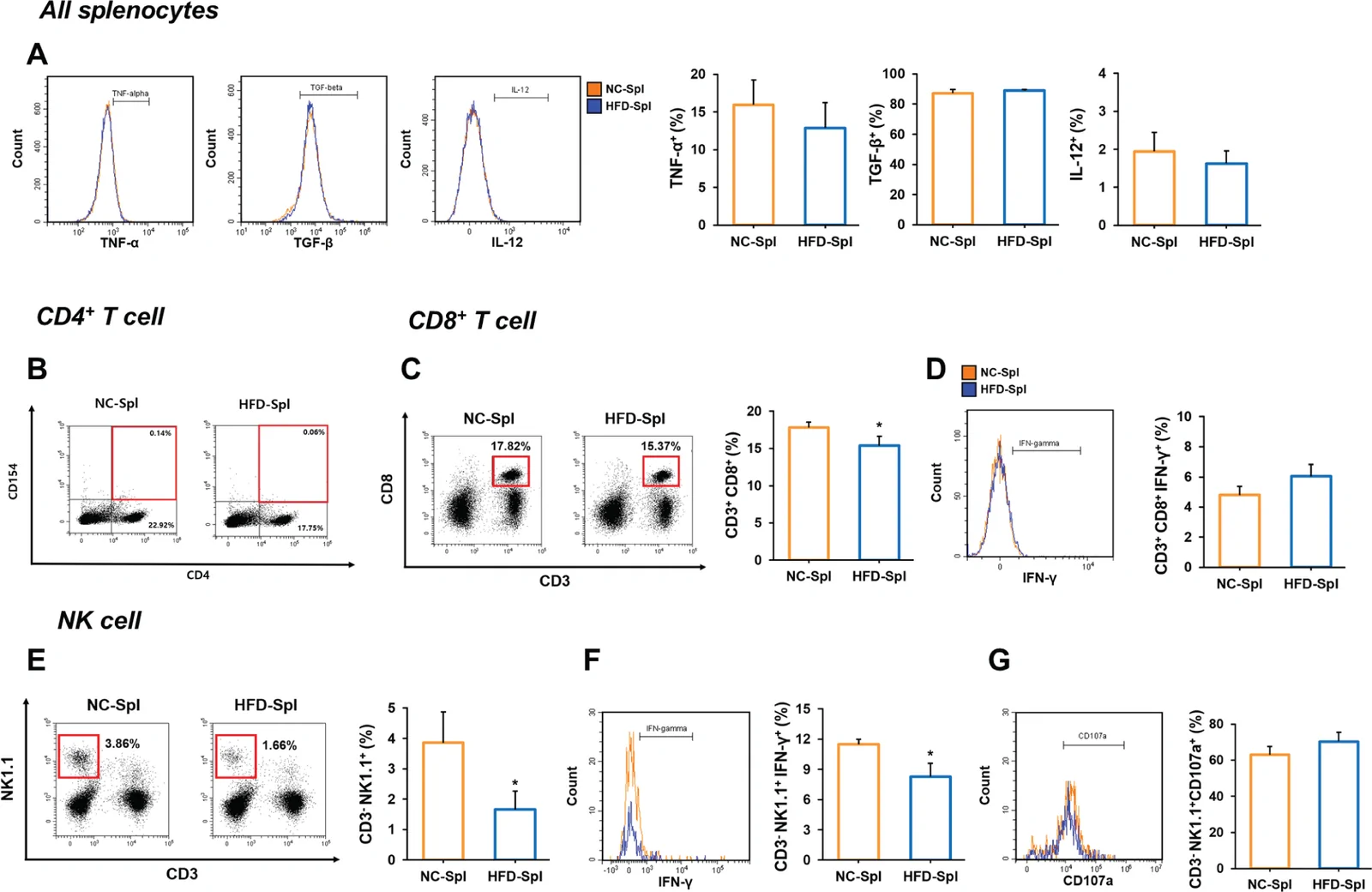
Effects of high-fat diet on splenocyte cytokine expression and immune cell composition in mice. (a) Representative histograms and quantification of TNF-α^+^, TGF-β^+^, and IL-12^+^ splenocytes from mice fed a normal chow diet (NC-Spl) or high-fat diet (HFD-Spl). Flow cytometry plots or histograms of (b) CD4^+^ T cells, (c) CD8^+^ T cells, (d) IFN-γ^+^ CD8^+^ T cells, (e) CD3^−^NK1.1^+^ cells, (f) CD3^−^NK1.1^+^IFN-γ^+^ cells, and (g) CD3^−^ NK1.1^+^ CD107a^+^ cells from NC-Spl or HFD-Spl. Data are expressed as the mean ± SD (*n* = 8). Statistical analyses were performed using Student’s *t*-test (*P < 0.05 vs. NC-Spl.).

To investigate the direct impact of obesity on systemic immune cell populations, we analyzed splenocytes from NC- and HFD-fed mice. Flow cytometric analysis of intracellular cytokine expression revealed no significant differences in the frequency of TNF-α^+^, TGF-β^+^, or IL-12^+^ splenocytes between the two groups (Fig. 1a), suggesting that HFD feeding does not significantly alter the overall cytokine profile of splenocytes.

Thereafter, we examined the distribution of T cell subsets. The proportion of CD4^+^ T cells was comparable between the NC and HFD groups (Fig. 1b). However, the abundance of CD8^+^ T cells was significantly reduced in the splenocytes of HFD-fed mice compared with that in the NC control group (*P* < 0.05; Fig. 1c). Functional assessment of CD8^+^ T cells indicated no significant difference in the frequency of IFN-γ-producing cells between the two groups (Fig. 1d). Analysis of NK cell populations revealed a significant reduction in CD3^−^NK1.1^+^ NK cells in HFD-fed mice compared with that in their NC-fed counterparts (*P* < 0.05; Fig. 1e). In addition, the proportion of IFN-γ-producing NK cells also significantly decreased in the HFD group (*P* < 0.05; Fig. 1f), while NK cell degranulation, assessed by CD107a expression, remained unchanged (Fig. 1g).

These findings suggest that HFD feeding reduces the abundance of cytotoxic lymphocytes, particularly CD8^+^ T and NK cells, while cytokine production remains largely unaffected.

### Splenocytes from HFD-fed mice exhibit impaired cytotoxic activity against Panc02 cells

**Fig. 2:**
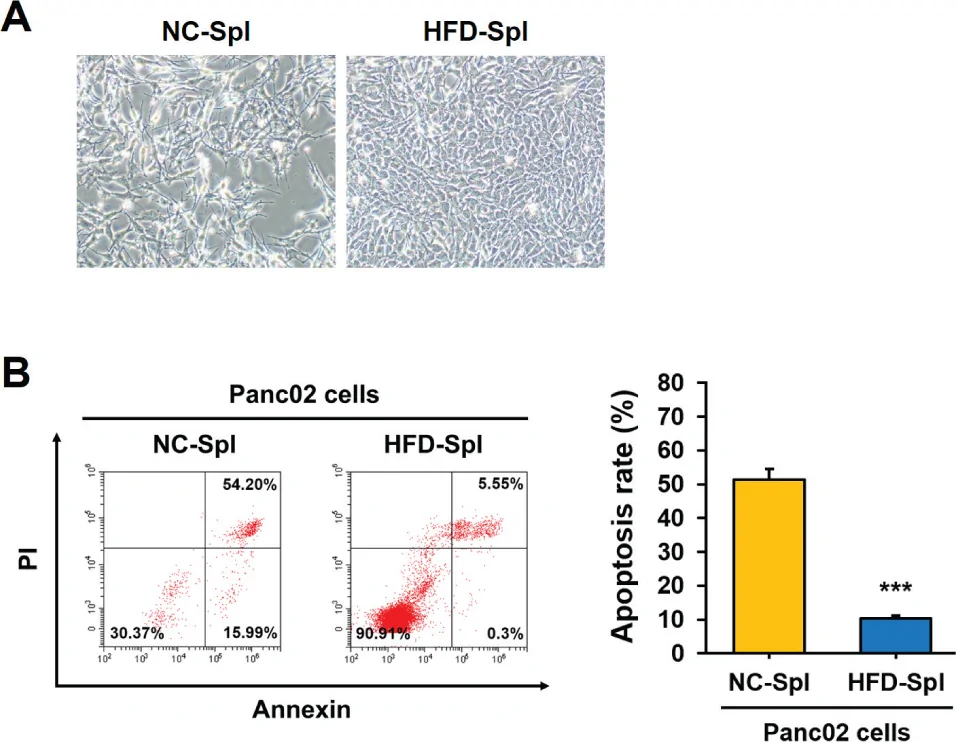
Effects of high-fat diet on splenocyte-mediated apoptosis in Panc02 cells. (a) Microscopic images of Panc02 cells following 42 h co-culture with NC-Spl and HFD-Spl. (b) Annexin V/PI flow cytometric analysis assessing apoptosis in Panc02 cells after co-culture with NC- or HFD-derived splenocytes. Data are expressed as the mean ± SD (*n* = 8). Statistical analyses were performed using Student’s *t*-test (**P < 0.01, ***P < 0.001 vs. NC-Spl.).

To investigate the impact of obesity on the cytotoxic function of immune cells, Panc02 cells were co-cultured with splenocytes isolated from NC- or HFD-fed mice. Microscopic images showed that Panc02 cells co-cultured with HFD-derived splenocytes exhibited higher cell density than those co-cultured with NC-derived splenocytes (Fig. 2a). Flow cytometric analysis utilizing annexin V/PI staining further revealed that NC-derived splenocytes markedly induced apoptosis in Panc02 cells, with over 50% of cells undergoing apoptotic death. In contrast, the apoptotic activity of the HFD-derived splenocyte group dramatically decreased to less than 10% (*P* < 0.001; Fig. 2b). These findings indicate that obesity significantly impairs the capacity of splenocytes to induce apoptosis in Panc02 cells.

### Splenocytes from HFD-fed mice diminish CD8^+^ T and NK cell abundance in co-culture with Panc02 cells

**Fig. 3:**
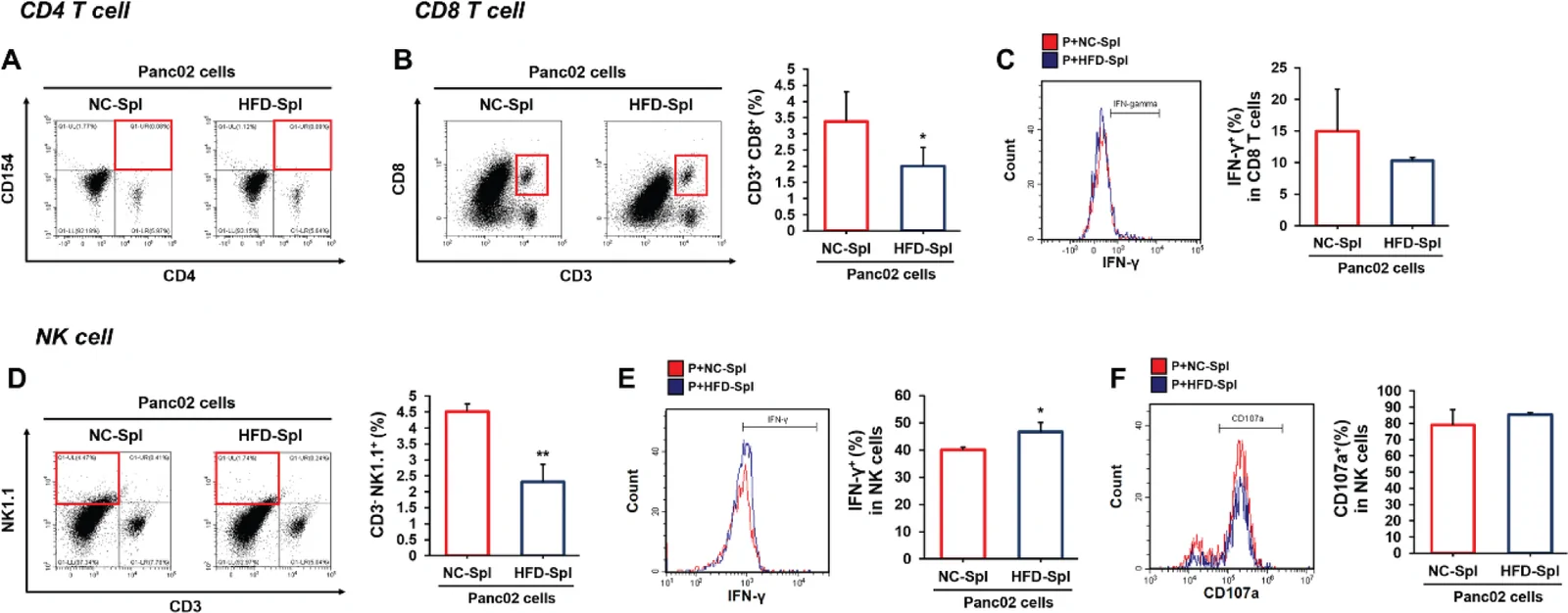
Effects of high-fat diet on immune cell composition and function in Panc02–splenocyte co-culture. Flow cytometry plots or histograms depict (a) CD4^+^ T, (b) CD8^+^ T, (c) IFN-γ^+^ CD8^+^ T, (d) CD3^−^ NK1.1^+^, (e) CD3^−^NK1.1^+^IFN-γ^+^, and (f) CD3^−^NK1.1^+^CD107a^+^ cells from Panc02 cells after co-culture with NC-Spl or HFD-Spl. Data are expressed as the mean ± SD (*n* = 8). Statistical analyses were performed using Student’s *t*-test (*P < 0.05, **P < 0.01 vs. NC-Spl.).

To examine the influence of obesity on the immune response during tumor–immune cell interactions, we analyzed splenocyte subsets following co-culture with Panc02 cells. The proportion of CD4^+^ T cells remained unchanged between NC- and HFD-derived splenocyte groups (Fig. 3a). Nonetheless, the frequency of CD8^+^ T cells significantly decreased in the HFD-derived splenocyte co-cultures compared with that in NC controls (*P* < 0.05; Fig. 3b), although no significant differences in the proportion of IFN-γ^+^ CD8^+^ T cells were observed (Fig. 3c).

Similarly, the frequency of CD3^−^NK1.1^+^ NK cells markedly decreased in HFD-derived splenocytes compared with that in NC-derived splenocytes (*P* < 0.01; Fig. 3d). Interestingly, functional profiling indicated a slight increase in IFN-γ-producing NK cells within the HFD group (*P* < 0.05; Fig. 3e), while degranulation activity (CD107a^+^ NK cells) remained unchanged (Fig. 3f). Taken together, these findings suggest that obesity reduces the abundance of cytotoxic lymphocytes in the tumor–immune microenvironment but does not significantly impair their functional activation, as evidenced by a compensatory increase in IFN-γ production by NK cells.

### Splenocytes from HFD-fed mice modulate immune checkpoint expression during co-culture with Panc02 cells

**Fig. 4:**
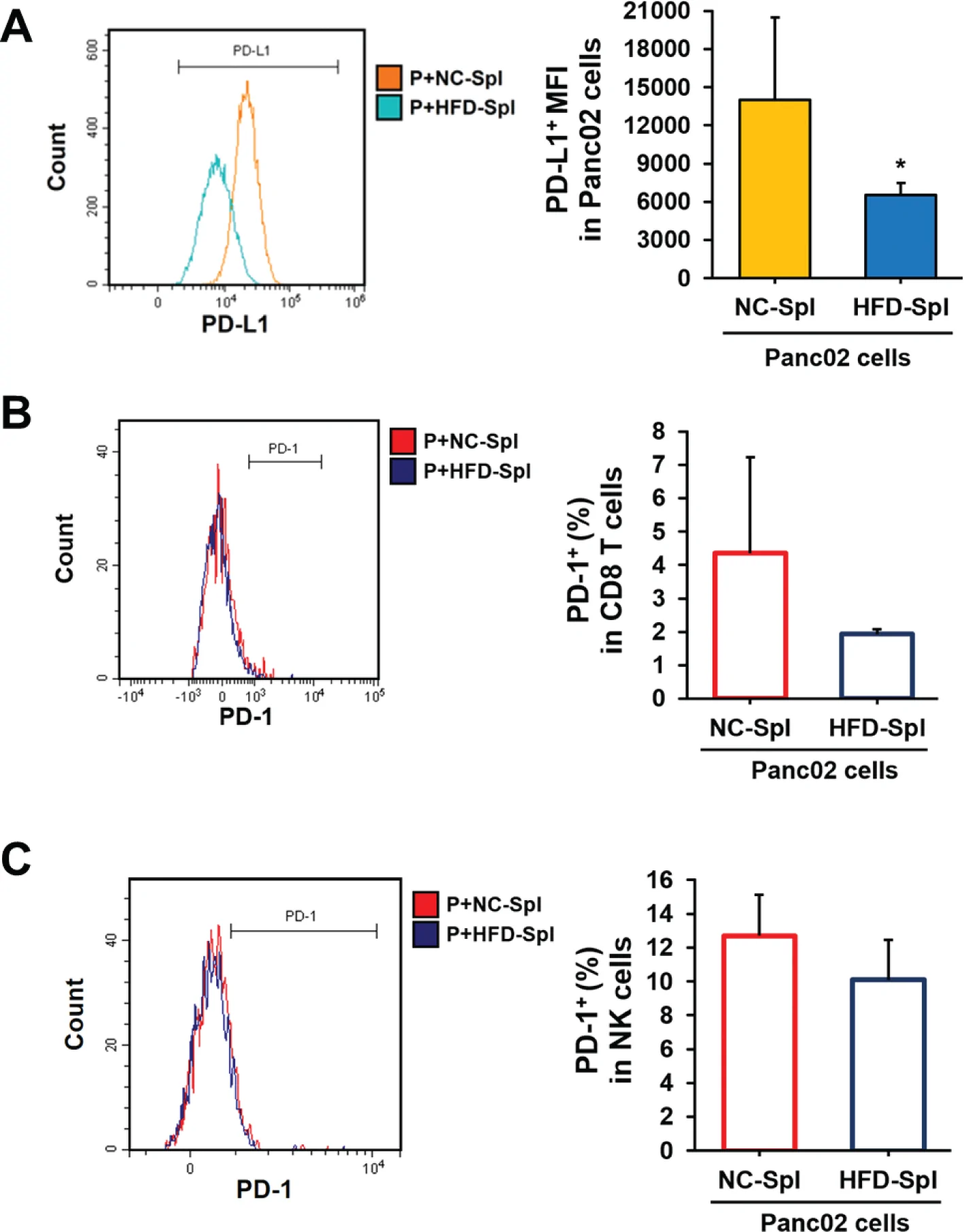
Effects of a high-fat diet on immune checkpoint expression in Panc02–splenocyte co-culture. Representative histograms and quantification of (a) PD-L1 expression in Panc02, (b) PD-1^+^ CD8^+^ T, and (c) PD-1^+^ NK cells. Data are expressed as the mean ± SD (*n* = 8). Statistical analyses were performed using Student’s *t*-test (*P < 0.05 vs NC-Spl.).

We assessed PD-L1 expression on Panc02 cells and PD-1 expression on splenic CD8^+^ T and NK cells following co-culture to determine whether obesity affects immune checkpoint pathways in the tumor–immune context. Unexpectedly, flow cytometry analysis revealed that Panc02 cells co-cultured with NC-derived splenocytes exhibited significantly higher levels of PD-L1 than those co-cultured with HFD-derived splenocytes (*P* < 0.05; Fig. 4a). Nevertheless, the proportions of PD-1^+^ CD8^+^ T cells and PD-1^+^ NK cells did not significantly differ between NC- and HFD-derived splenocyte co-cultures (Fig. 4b, c). Collectively, these findings suggest that the inhibition of apoptotic activity in Panc02 cells induced by HFD-derived splenocytes is not attributable to altered PD-1 expression on CD8^+^ T or NK cells.

## Discussion

Obesity has long been associated with the progression of PDAC through systemic metabolic alterations, including chronic inflammation, insulin resistance, and altered adipokine signaling (10–13). While these mechanisms are well established, the immunological implications of obesity, particularly regarding the interactions between immune and tumor cells in PDAC progression, have yet to be fully elucidated. In this study, we demonstrated that HFD feeding altered the splenocyte composition by decreasing the proportion of cytotoxic lymphocytes, which in turn reduced apoptosis in Panc02 cells. These findings provide evidence that obesity attenuates immune-mediated tumor suppression, thereby contributing to the tumor-permissive environment observed in obesity.

We first demonstrated that obesity significantly alters systemic immune cell composition. Splenocytes from HFD-fed mice displayed a reduced abundance of cytotoxic lymphocytes, specifically CD8^+^ T and NK cells, compared with those from NC-fed controls. Notably, the cytokine-producing capacity of these cells was largely preserved. CD8^+^ T and NK cells are essential for anti-tumor immunity, employing perforin/granzyme-mediated killing and cytokine secretion (14–17). This finding suggests that while obesity may not entirely suppress immune activation, it diminishes the pool of effector immune cells available for tumor surveillance. Such quantitative suppression of cytotoxic lymphocytes has been documented in both preclinical and clinical models of obesity, where reduced CD8^+^ T cell infiltration and NK cell dysfunction correlate with accelerated tumor growth (18–21).

Building on this observation, our co-culture model revealed that HFD-derived splenocytes exhibited markedly impaired cytotoxic activity, resulting in decreased apoptosis of Panc02 cells. This functional impairment was accompanied by a reduction in the frequency of both CD8^+^ T and NK cells during co-culture, suggesting that obesity compromises anti-tumor immunity primarily through quantitative rather than qualitative alterations in cytotoxic lymphocytes. However, because splenocytes were used as the primary immune cell source, the present model may not fully capture the complexity and cellular heterogeneity of the pancreatic TME.

Interestingly, we found that PD-1 expression on CD8^+^ T and NK cells remained unchanged, while PD-L1 expression on Panc02 cells was downregulated when co-cultured with HFD-derived splenocytes, despite the reduced abundance of cytotoxic lymphocytes. The PD-1/PD-L1 immune checkpoint axis is a key regulator of tumor immune evasion and significantly influences responses to immunotherapy. PD-L1 upregulation in the TME is widely recognized as a mechanism of adaptive immune resistance, enabling tumor cells to evade immune-mediated destruction (22). Conversely, PD-L1 positivity may also arise from immune response-induced expression, reflecting active immune engagement. Therefore, PD-L1 serves as a dual-purpose biomarker whose biological significance is context-dependent (22–24). In this context, the decreased PD-L1 expression observed in our study likely reflects weakened immune activation linked to obesity rather than the alleviation of checkpoint suppression.

Overall, our findings suggest that obesity impairs anti-tumor immunity in PDAC by reducing the abundance of cytotoxic lymphocytes. This reduction diminishes immune pressure on tumor cells and limits PD-L1 induction. The resulting immune landscape reveals a predominance of immune deficiency over immune exhaustion in obesity-associated PDAC. This observation supports the development of therapeutic strategies aimed at restoring the activity and recruitment of cytotoxic immune cells to enhance anti-tumor immunity in patients with obesity and pancreatic cancer. Further research is warranted to explore how metabolic interventions, immune cell expansion therapies, or combination strategies can improve anti-tumor immunity in obesity-associated PDAC.

## Conclusion

This study demonstrates that obesity impairs anti-tumor immunity in PDAC by altering immune cell composition and reducing apoptotic activity. These findings suggest that obesity primarily compromises anti-tumor immunity through a quantitative loss of effector immune cells rather than functional exhaustion. Targeting the restoration of cytotoxic lymphocyte composition may represent a promising strategy to normalize immune balance and improve therapeutic outcomes in obesity-associated PDAC.
